# Effect of UV-B Radiation on Flavonoids and Phenols Accumulation in Tempisque (*Sideroxylon capiri* Pittier) Callus

**DOI:** 10.3390/plants11040473

**Published:** 2022-02-09

**Authors:** Karina E. Martínez-Silvestre, José Alfredo Santiz-Gómez, María Celina Luján-Hidalgo, Nancy Ruiz-Lau, Yazmin Sánchez-Roque, Federico A. Gutiérrez-Miceli

**Affiliations:** 1 Tecnológico Nacional de México, División de Estudios de Posgrado e Investigación, Instituto Tecnológico de Tuxtla Gutiérrez, Carretera Panamericana Km 1080, Tuxtla Gutiérrez 29050, Chiapas, Mexico; M14270401@tuxtla.tecnm.mx (K.E.M.-S.); jose.sg@tuxtla.tecnm.mx (J.A.S.-G.); maria.lh@tuxtla.tecnm.mx (M.C.L.-H.); 2 Cátedra CONACYT—Tecnológico Nacional de México-Instituto Tecnológico de Tuxtla Gutiérrez, Carretera Panamericana Km 1080, Tuxtla Gutiérrez 29050, Chiapas, Mexico; nancy.rl@tuxtla.tecnm.mx; 3 Dirección de Ingeniería Agroindustrial, Universidad Politécnica de Chiapas, Carretera Tuxtla Gutiérrez-Portillo Zaragoza Km 21+500, Colonia Las Brisas, Suchiapa 29150, Chiapas, Mexico; ysanchez@ia.upchiapas.edu.mx

**Keywords:** tempisque, callus, UV-B radiation, phenols, flavonoids

## Abstract

Tempisque (*Sideroxylon capiri* Pittier) is classified as a threatened species and has been reported with a high content of phenols and flavonoids in the leaves. The use of abiotic elicitors such as radiation has been reported due to the changes it produces in the metabolism of plants by activating their defense mechanisms and increasing the biosynthesis of bioactive compounds with antioxidant capacity such as phenols and flavonoids. Therefore, the aim of this work was to evaluate the effect of UV-B radiation on growth parameters and the synthesis of bioactive compounds in in vitro culture of tempisque callus. For the callus induction, we used thidiazuron (TDZ) and 2,4-dichlorophenoxyacetic acid (2,4-D) at 0, 0.5 and 1 mg/L. Calluses were exposed to UV-B radiation (0, 1, 2, 3 and 4 h/day) for two and four weeks. The highest callus formation index was obtained with TDZ and 2,4-D at 1 mg/mL. The greatest increase in the concentration of phenols and flavonoids was detected in the fourth week with 4 h of exposure per day. The highest concentrations of quercetin (230 µg/g dry weight), kaempferol (235 µg/g dry weight) and gallic acid (240 µg/g dry weight) were found in callus obtained from leaves explants.

## 1. Introduction

The tempisque (*Sideroxylon capiri* Pittier) is a timber tree that grows up to 40 m high, and it is distributed from Mexico to Panama [1]. It is considered native to Mexico and is used mainly by the peasant population for the construction of homes, as fuel, for foraging, and for ornamental purposes. However, in most cases, the plant used by the population is obtained directly from forests, jungles or secondary vegetation without there being a sustainable management or cultivation of this species [2]. Thus, the tempisque is categorized as a threatened species in Mexico, according to the Ecological Standard NOM-059-SEMARNAT-2010 [3]. In addition, the seeds germination rate, lower than 30%, is one aspect that prevent its spread due to the fact that it has recalcitrant seeds, which reduces the viability index [1,2].

Additionally, the tempisque is a tree whose leaves and fruits are edible and are also used as a condiment in food and for some kidney diseases and it has been qualitatively determined that the leaf extracts contain secondary metabolites such as phenols and flavonoids mainly, but steroids and tannins also have been determined [4]. This species could be a good source of secondary metabolites with important properties for the pharmaceutical and food industry due to the leaf extracts being practically non cytotoxic, so their use could be safer in relation to the absence of saponins and alkaloids since toxic properties have been attributed to these compounds [4]. In the same way, leaf extract of tempisque has allowed potassium nanoparticle synthesis due to the secondary metabolites it contains, and these phytonanoparticles have shown effectiveness versus pathogenic microorganisms such as *Bacillus cereus*, *Enterobacter aerogenes*, *Fusarium solani* and *Botrytis cinerea* [5].

Plant secondary metabolites are valuable sources for traditional medicine, food industry, cosmetics and pharmaceuticals [6]. Phenolic compounds have important functional properties such as antioxidant, anticancer and anti-inflammatory capacities, among others [7]. Gallic acid has pharmacological and cosmetic applications as it repairs the barrier function of the epidermis to treat damage caused by conditions such as Crohn’s disease [8], while flavonoids such as quercetin and kaempferol are in great industrial demand for their various biotechnological applications [9]. Kaempferol is used for the treatment of heart disease and oxidative stress, for the attenuation of diabetic nephropathy and in the treatment of respiratory diseases [10,11,12,13], while quercetin has been used as an antioxidant drug, in the treatment of erythroleukemia and against cervical cancer due to its high cytotoxicity against cancer cells [14,15,16]. Several studies reported that when medicinal plants were exposed to abiotic stresses such as drought, heavy metals, and nutrient deficiency, they showed changes in their physiology, morphology and the biosynthesis of plant secondary metabolites [6,17]. One of the most relevant abiotic stresses for plants is the UV-B radiation which has significant effects on defense-related biosynthesis of secondary metabolites [18] and it can be used as a simple and environmentally friendly method for increasing the content of bioactive components.

Some research studies have shown that UV-B is a strategy that has been used to induce terpenoid synthesis in *Ocimum basilicum* [19]. In the same way, UV-B radiation increased essential oil levels and phenols content in *Acorus calamus* L. [20], while in *Morus alba* L., irradiation in vitro with UV-B induced the production of bioactive secondary metabolites in leaves [21]. Additionally, some studies indicated that UV-B radiation significantly promotes the synthesis of secondary metabolites and antioxidant activity, as well as the content of the main bioactive components in *Prunella vulgaris* L. [22,23]. In *Echium orientale*, it has been reported that callus extracts contain higher amount of total phenolics and flavonoids and the phenolic compounds than shoot extracts and that UV-B irradiation increases the antioxidant activity and total flavonoid and phenols content [24].

To the best of our knowledge, no report is available in the literature concerning the quantitative profiles of secondary metabolites in tempisque, and a potential alternative for increasing the accumulation of secondary metabolites such as phenols and flavonoids could be callus culture and the use of UV-B radiation. Therefore, the aim of this research was to evaluate the responses induced by UV-B radiation on growth and the concentration of phenols and flavonoids at different exposure times in tempisque (*Sideroxylon capiri* Pittier) callus culture.

## 2. Results

### 2.1. Explant Disinfection and Callus Induction

Explants of leaf, stem and root had different responses in disinfection percentage. The highest disinfection percentages were obtained in leaves with 95% (*p* ≤ 0.05) and later stem with 70%, and root explant disinfection was not achieved. While the percentage of survival of the explants after disinfection was 90% in the leaf explants and 94% in the stem explants, in the root explants it was not possible to determine survival as they were completely contaminated.

**Figure 1:**
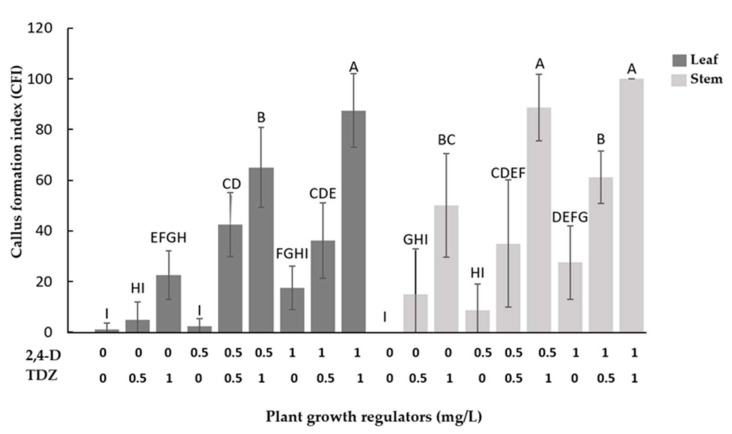
Effect of 2,4-D and TDZ on callus formation index (CFI) after 40 days of growth. Bars which do not share a common letter are significantly different (*p* ≤ 0.05).

With respect to the callus formation index (CFI), it is observed that when plant growth regulators are used individually, the callus formation index is low compared to when 2,4-D and TDZ are used in combination. In stem explants, the use of 2,4-D (1 mg/L) + TDZ (1 mg/L) shows a better CFI compared to other results, while leaf explant had a better CFI with 2,4-D (0.5 or 1 mg/L) + TDZ (1 mg/L). In both explants, it is observed that the individual use of TDZ increases the CFI compared to the individual use of 2,4-D (Figure 1).

**Figure 2:**
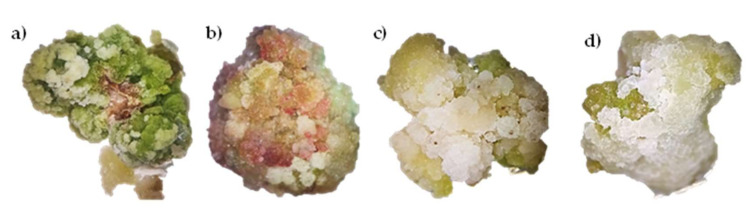
Callus obtained from tempisque explants after 40 days of incubation, (**a**) callus obtained from leaf with 1 mg/L of 2,4-D and 1 mg/L of TDZ, (**b**) callus obtained from leaf with 0.5 mg/L of 2,4-D and 1 mg/L of TDZ, (**c**) callus obtained from stem with 1 mg/L of 2,4-D and 1 mg/L of TDZ, (**d**) callus obtained from stem with 0.5 mg/L of 2,4-D and 1 mg/L of TDZ.

The type of explant, either leaf or stem, and growth regulators 2,4-D and TDZ had an effect on the type and morphology of tempisque callus generated after 40 days of incubation and growth. The callus obtained from leaf explants were compact with green coloration and in some cases with orange-violet tones, while the callus obtained from stem explants were friable with white-yellow coloration. Calluses obtained without TDZ were phenolizated, whereas calluses obtained with TDZ presented green coloration, without phenolized areas (Figure 2). Therefore, it can be assumed that TDZ plays a very important role in the induction of callogenesis in tempisque.

### 2.2. Effect of UV-B Radiation on Callus Growth

The results of the exposure time under UV-B in callus obtained from different explants on the fresh weight showed that the general trend was the gradual increase in callus weight with increasing exposure times compared to the control (without UV-B radiation) both in callus obtained from leaf and stem explants. Therefore, the time of 4 h of exposure to UV-B radiation was statistically significant (*p* ≤ 0.05) in the increase in the fresh weight of callus obtained from both leaf and stem explants during two weeks of irradiation while after 4 weeks of irradiation at a time of 4 h of exposure, it only showed a statistically significant difference in the increase in the fresh weight of the calluses obtained from leaf explants.

Regarding the effect of the explant, at all times of exposure, callus obtained from leaf explants had a greater increase in fresh weight with respect to the callus obtained from stem explants in a period of two weeks of irradiation, while in a period of 4 weeks of irradiation, only the callus obtained from leaf explants showed a linear increase in fresh weight. For the interactions in the two-week period, the highest significant values for fresh weight were 907.3 and 894 mg with 4 h of daily exposure to UV-B light in calluses from leaf and stem explants, respectively.

However, for the four-week period with 4 h of daily exposure in calluses from leaf explants, an increase in fresh weight was observed with 1289.4 mg, but in calluses obtained from stem explants, a decrease in fresh weight was observed with 695.8 mg, which is less than the 847.1 mg obtained for the control (Table 1), suggesting the use of callus obtained from leaf explants to induce a higher fresh weight using UV-B radiation.

In the same way, the effect of UV-B radiation on the dry weight of the callus culture showed a behavior similar to that obtained in fresh weight. The general trend during two weeks of exposure was the increase in the dry weight of the callus during each exposure time, both in callus obtained from leaves and stems explants. The highest dry weight was 83.86 mg in callus obtained from leaf explants after 4 h of exposure and was statistically significant (*p* ≤ 0.05) for this exposure period, while in calluses obtained from stem explants, the highest dry weight was 70.2 mg after 4 h of exposure.

On the other hand, for the period of four weeks of exposure, the highest dry weight was 149.3 mg in the calluses obtained from leaf explants during four hours of exposure, and this was statistically significant (*p* ≤ 0.05) with respect to the other exposure times in the callus obtained from both leaf and stem explants. In the callus obtained from stem explants, it was observed that after one hour of exposure, there is no statistically significant increase in the other exposure times (Table 2), which suggests that in this period and long exposure times, oxidation and death of the callus begins.

Regarding the growth index, it was observed that at four hours of exposure time during the two-week period, the growth index was the highest both in the callus obtained from leaf explants and in the callus obtained from stem explants, the time of four hours being statistically significant (*p* ≤ 0.05) compared to the other treatments.

On the other hand, for the four-week period, it was observed that in the callus obtained from leaf explants, the exposure time of four hours showed a statistically significant difference (*p* ≤ 0.05) with respect to the other treatments and with the two-week period in the same exposure time, while in the callus obtained from stem explants, the highest growth index was observed at three hours of exposure and was reduced at four hours of exposure (Table 3).

These results show that in the callus obtained from leaf explants, the longer the time and the period of exposure, there will be greater growth of callus, while in the callus obtained from stem explants at periods greater than 3 weeks and times of more than four hours, the growth is reduced.

### 2.3. Effect of UV-B Radiation on Morphology and Phenolization of Callus

In this study, it was observed that UV-B radiation had an effect on the morphology and phenolization of calluses. The callus obtained from leaf explants irradiated for two weeks increased its green hue and presented a more compact and spongy structure as the exposure time to UV-B radiation increased, while the callus obtained from stem explants increased its yellow-cream hue and presented a more compact and spongy structure with increasing exposure time.

On the other hand, when the callus was irradiated for a period of four weeks, it was observed that from the time of one hour of exposure, the morphology of the callus was more compact and spongy and it maintained its green hue and in all the irradiation times areas were generated. Brown color and callus phenolization were observed in most of the callus surfaces, while calluses obtained from stems irradiated in the same period of time increased their yellow-cream hue in all exposure times, but also presented areas brown in color and phenolized surface areas.

**Figure 3:**
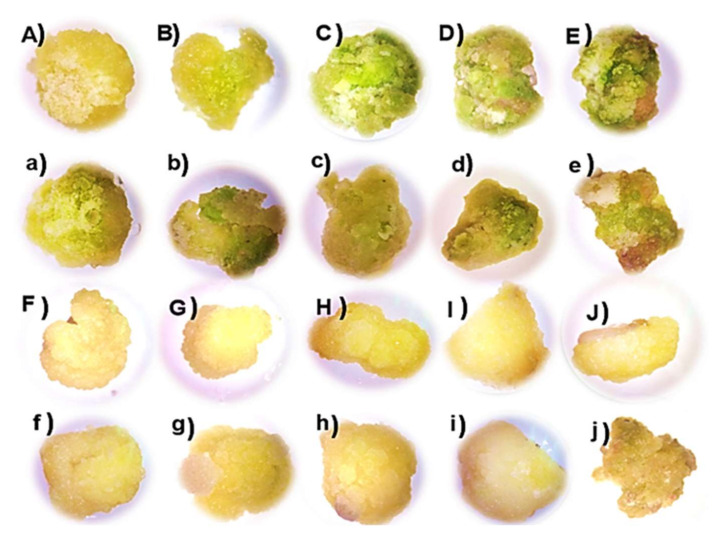
Morphology and phenolization of callus irradiated with UV-B light. (**A**) Callus obtained from leaf explants (control), (**B**) callus obtained from leaf explants with 1 h of irradiation, (**C**) callus obtained from leaf explants with 2 h of irradiation, (**D**) callus obtained from leaf explants with 3 h of irradiation, (**E**) callus obtained from leaf explants with 4 h of irradiation, (**F**) callus obtained from stem explants (control), (**G**) callus obtained from stem explants with 1 h of irradiation, (**H**) callus obtained from stem explants with 2 h of irradiation, (**I**) callus obtained from stem explants with 3 h of irradiation, (**J**) callus obtained from stem explants with 4 h of irradiation. Capital letters are for calluses with two weeks of exposure and lowercase letters with four weeks of exposure.

With respect to the phenolization, partial phenolization can be seen in the four exposure times evaluated in calluses obtained from both leaf and stem explants. The highest phenolization occurred in calluses irradiated for four hours a day in a period of four weeks, in calluses obtained from both leaf and stem explants but mainly in calluses obtained from stem explants (Figure 3).

### 2.4. Total Phenol and Flavonoid Content in Callus Irradiated with UV-B Light

The effect of UV-B radiation on the concentration of total phenols showed a general trend that an increase in the concentration of phenols can be seen with increasing exposure times, presenting a statistically significant difference (*p* ≤ 0.05) in two, three and four hours of exposure compared to the control.

Regarding the type of explant, the callus obtained from leaf explants presented a higher concentration of total phenols with statistical significance (*p* ≤ 0.05) compared to the callus obtained from stem explants. The highest concentration of total phenols was obtained in extracts of callus obtained from leaf explants with four hours of daily irradiation for a period of four weeks with 12.2 mg per gram of callus dry weight (Table 4).

Regarding the content of total flavonoids, it was observed that during the period of two weeks of irradiation, all the exposure times evaluated increased the concentration of flavonoids with a statistically significant difference (*p* ≤ 0.05) with respect to the control, in calluses obtained from both leaf and stem explants, but the callus obtained from leaf explants presented the largest amount of total flavonoids at four hours of exposure. On the other hand, for the period of four weeks of irradiation, it was observed that the calluses obtained from leaf and stem explants produced a greater amount of flavonoids compared to the treatments irradiated for two weeks, and in the same way, the callus obtained from leaf explants with four hours of exposure presented the highest amount of flavonoids (Table 5).

### 2.5. Effect of UV-B Radiation on Gallic Acid, Quercetin and Kaempferol Concentrations

**Figure 4:**
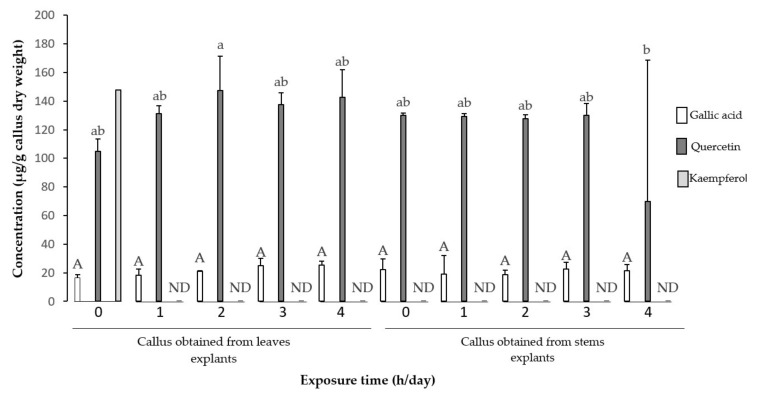
Effect of time of exposure to UV-B light on the concentration of gallic acid, quercetin and kaempferol in calluses with 2 weeks of exposure. Upper case letters indicate significance for gallic acid, lower case letters indicate significance for quercetin and numbers indicate significance for kaempferol with 1 being the highest significant level (*p* ≤ 0.05). ND = not detected.

The effect of UV-B radiation over a period of two weeks showed that there was no significant difference in the content of gallic acid in calluses obtained from leaf and stem explants at the exposure times evaluated. For quercetin, it had a significant difference (*p* ≤ 0.05) between four hours of exposure in calluses obtained from stem explants and two hours in calluses obtained from leaf explants. Kaempferol could not be detected at all exposure times on UV-B radiation (Figure 4).

**Figure 5:**
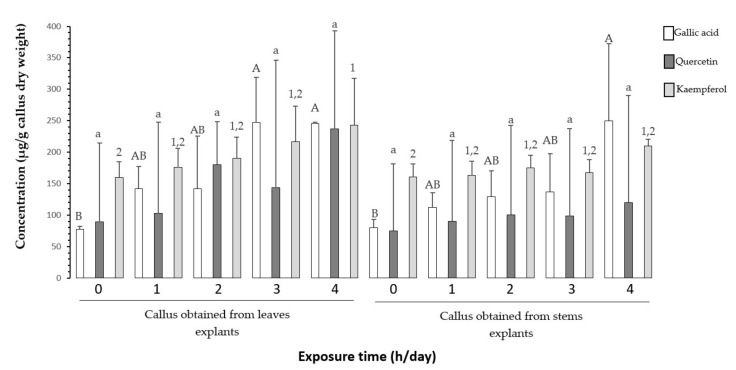
Effect of time of exposure to UV-B light on the concentration of gallic acid, quercetin and kaempferol in calluses with four weeks of exposure. Capital letters indicate significance for gallic acid, lower case letters indicate significance for quercetin, and numbers indicate significance for kaempferol, with 1 being the highest significant level (*p* ≤ 0.05).

For the four-week period, the results showed that the highest concentrations of quercetin and gallic acid obtained for this period were with 4 h of daily exposure in calluses obtained from leaf explants, with a statistically significant difference (*p* ≤ 0.05) only for gallic acid compared to the control. Regarding the concentration of kaempferol in the irradiated treatments, a significant statistical difference was observed in the exposure time of 4 h a day in calluses obtained from both leaf and stem explants with respect to the control (Figure 5). These results suggest that these three compounds participate in the metabolic antioxidant defense against the stressful conditions induced by UV-B light.

## 3. Discussion

### 3.1. Explant Disinfection

One of the main requirements for the establishment of an in vitro culture is asepsis; for this reason, the first option for obtaining aseptic material is the disinfection protocol. However, this is not a simple process in all species; it depends on the heterogeneity, size, viability and latency, which are strongly dependent on the genotype. In tempisque, it has been described that the hardness of the seed coat makes both the disinfection and germination processes difficult [2]. Similarly, it has been reported that the recalcitrance of tempisque seeds presents serious limitations for their storage for propagation purposes. This is due to the rapid loss of viability [2,25]. The results in the disinfection of the explants are relevant because they allowed us to obtain disinfection percentages of 70 to 95% and high survival rates from 90 to 94% in explants from stems and leaves, respectively, which represents an alternative in obtaining plant material for its in vitro management. Similar results have been obtained in explants of *Passiflora caerulea* and *Passiflora quadrangularis* using three concentrations of NaClO (0, 5, 10%) at different exposure times (10, 15 and 20 min), obtaining the highest percentage of disinfection with NaClO at 5% (*v*/*v*) and 15 min of exposure [26]. The results contained in this research allow us to point out that a higher concentration is not always the best alternative to increase the disinfection percentages, so that in the case of tempisque explants, an increase in the exposure time can allow better results, avoiding the fact that the concentrations and disinfection times are not toxic to the explant and subsequently allow the regeneration of the explants to induce any process at the in vitro level.

### 3.2. Callus Induction

Regarding callogenesis, it has been demonstrated that a combination of cytokinin and auxin induces callus formation and that the balance between cytokinin and auxin determines the state of differentiation and dedifferentiation of the callus [27]. Additionally, recalcitrant woody species had a great response to TDZ due to their high activity, similar to cytokinins, presenting an even better response than natural endogenous cytokinins [28]. TDZ effects have been discussed in detail by many researchers, given that despite being classified mainly as cytokinin, it also presents auxin activities promoting cell division, growth and differentiation [29,30]. This dual hormonal behavior of TDZ favored the development of callus when used individually in the culture medium and increased this effect when used together with 2,4-D at the same concentrations as could be observed in the results in both leaf and stem explants (Figure 1). In explants of arboreal peony (*Paeonia ostii* ‘Fengdan’), the highest index of callus formation has been reported with 0.5 mg/L of TDZ and 0.5 mg/L of 2,4-D, which is attributed to the signaling function of auxins (2,4-D), to promote cell cycle reentry and reacquisition of cell proliferative competence, which makes it the key regulator for the induction of cell dedifferentiation [29], which, when combined with the cell growth capacity offered by TDZ and the protection by inhibiting cytokinin oxidase, greatly improves callus proliferation [31].

### 3.3. Effect of UV-B Radiation on Callus Growth

In this research, the general trend was the gradual increase in fresh weight with increasing exposure times and exposure periods compared to the control without radiation, in calluses obtained from both leaf and stem explants (Table 1). It has been shown that the use of radiation not only inhibits but can also promote plant growth and that this positive or negative effect largely depends on the species, intensity and radiation times to which the study model is subjected [32]. In the same way, the effect of UV-B radiation on callus culture and cell suspension of *Echinacea purpurea* has been evaluated, achieving an increase in fresh biomass in irradiated treatments compared to non-irradiated ones due to the increase in the biosynthesis capacity of secondary metabolites with the antioxidant capacity that each species possesses for its adaptation to adverse conditions [33]. The competence of plant cells to maximize their growth depends on their ability to detect and respond to the amount of light. The results obtained in tempisque callus showed that all the exposure times at UV-B radiation promoted the increase in fresh weight.

The increase in fresh weight in calluses after two weeks of irradiation (Table 1) shows that water accumulation in callus possibly occurs due to the neutralization of ROS by phenolic and flavonoid compounds. This same behavior is observed in the growth index that depends on the fresh weight of the callus. Therefore, fresh weight and growth rate increase with time and UV-B irradiation period, but in calluses from stem explants after 4 h of irradiation over a period of 4 weeks, a decrease in fresh weight was observed. However, regarding the dry weight of the callus (Table 2), it is possible to see that the general behavior is the increase in dry weight with respect to time and the period of exposure to UV-B. Therefore, correlating these results, it is possible to affirm that UV-B radiation induces a proliferation and active growth of cells in tempisque callus and not only the accumulation of water, mainly in calluses obtained from leaf explants.

In this sense, plants contain a varied number of photoreceptors capable of capturing different wavelengths due to the variability of the electromagnetic spectrum [34,35,36]. The capture of UV-B light occurs by the UVR8 photoreceptor [37]. After UV-B perception, the UVR8 receptor monomerizes and interacts with COP 1, which is a signaling regulator, thus inhibiting the degradation of HY5 mediated by COP 1. HY5 accumulates after exposure to UV-B rays and regulates the expression of the transport and signaling genes for auxins and gibberellic acid. At low UV-B irradiations, gibberellic acid in its active form inactivates the DELLA proteins, which in turn allows the PIF to bind to the auxin biosynthesis promoter and the Aux/AIA genes and positively and negatively regulate its expression [38,39,40]. The importance of auxin biosynthesis lies in its participation for entry into the cell cycle [28]. According to the growth index obtained in the tempisque callus (Table 3), it is possible to observe that the incidence of UV-B radiation was taken advantage of by the photoreceptors and convert this type of radiation into biomass as occurs with an increase in fresh and dry weight and the growth index mainly in calluses obtained from leaf explants.

### 3.4. Effect of UV-B Radiation on the Morphology and Phenolization of Callus

With respect to the morphology of the callus, it was observed that the use of individual TDZ generated compact and spongy calluses, while the combination of TDZ with 2,4-D induced less-compact, spongy, and in some cases friable calluses in calluses obtained from both leaf and stem explants, TDZ induced calluses with less phenolization compared to the treatments where only 2,4-D was used (Figure 2), so these results show that the use of TDZ is crucial in the induction of callogenesis in tempisque. On the other hand, the phenolization and the physiological and biochemical mechanisms in callus have been extensively studied [41,42]. Phenolization is defined as the oxidation generated by free radicals of different cellular components, as well as the oxidation of phenolic compounds catalyzed by the enzyme polyphenol oxidase that catalyzes the oxidation of phenols to quinones, which in turn polymerize to form brown pigments. These chemical species are very prone to react, generating damage and even cell death [42,43,44], as could be observed in calluses obtained from leaf and stem explants irradiated for four hours over four weeks (Figure 3).

### 3.5. Total Phenol and Flavonoid Content in Calluses Irradiated with UV-B Light

In relation to the effects of UV-B radiation on the primary and secondary metabolism, it has been reported that the metabolic changes lead to morphological changes for the protection against this abiotic factor, and within protection mechanisms is the production of compounds with antioxidant capacity such as phenols and flavonoids [45,46,47,48]. These compounds counteract the damage generated by reactive oxygen species that are signaling agents of stressful conditions such as radiation. In addition, these compounds have the ability to absorb photons with the wavelength of the ultraviolet region, which is why they function as solar filters [49,50]. The concentration of total phenols in tempisque callus increased with the exposure times and were higher during four weeks of exposure, mainly in the callus obtained from leaf explants (Table 4). In this study, the total phenolic contents in calluses of *S. capiri* under UV-B radiation were higher than those of control treatment. Similar results were obtained in highbush blueberry leaves [51], *Echinacea purpurea* [33] and *Prunella vulgaris* L. [22]. Some studies have shown that phenolic compounds absorb in the UV-B electromagnetic region (280–320 nm) and these compounds function to protect against UV-B radiation [52,53]. Our results allow the suggestion that increasing the total phenolic contents induced by UV-B radiation could have beneficial effects on the callus growth.

In the same way, flavonoid contents in calluses of *S. capiri* increased under UV-B radiation (Table 5). Flavonoids could help maintain photosynthetic pigment levels and normal photosynthetic activity [54]. Similar results have been reported in previous studies with leaves of *Ginkgo biloba* L. [18] and *Taxus chinensis* [55]. Therefore, our results indicated that increased flavonoids could protect against UV-B radiation. However, the concentration of total flavonoids was lower than the concentration of total phenols. Therefore, the increase in the concentration of total phenols and flavonoids in the irradiated treatments compared to the control can be attributed to an increase in the defense mechanism of the tempisque cells to counteract the harmful effects that can cause the exposure to UV-B radiation.

### 3.6. Effect of UV-B Radiation on Gallic Acid, Quercetin and Kaempferol Concentrations

The roll of quercetin, gallic acid, and kaempferol in UV-B light defense has been widely reported [56,57]. These compounds have a high antioxidant activity due to the structure that each one possesses. The higher the number of hydroxyl groups and double bonds, the higher it will be [7,58]. Unlike the behavior presented for total phenols and flavonoids, a lower statistical significance is observed for the quantified compounds in the four evaluated exposure times. After two weeks, only gallic acid, quercetin and kaempferol were quantified in the control treatment, while in all other treatments, only gallic acid and quercetin were quantified (Figure 4). However, after four weeks of irradiation in all treatments, the presence of gallic acid, quercetin and kaempferol was determined mainly in the calluses obtained from leaf explants (Figure 5). Under stress conditions, the flavonoid enzyme 3’-hydroxylase catalyzes the conversion of dihydrokaempferol to dihydroquercetin, which are precursors of kaempferol and quercetin, respectively [59]. From this perspective, it can be inferred that in the second week of exposure, kaempferol biosynthesis was interrupted in irradiated callus, redirecting the route to quercetin biosynthesis, which has greater antioxidant capacity. However, by increasing the exposure period to 4 weeks, it is possible that the biosynthesis pathway is redirected to the accumulation of kaempferol to reduce or avoid cell damage, and for this reason it was possible to quantify it in the treatments with four weeks of irradiation. UV-B radiation can regulate the activity of various enzymes that catalyze the synthesis of phenolic compounds [60,61,62]. Consequently, enzymatic activity of key enzymes of the phenyl propanoid pathway and shikimic acid, as well as the determination of gene expression could explain more precisely the behavior of phenolic metabolism in tempisque callus.

## 4. Materials and Methods

### 4.1. Plant Material and Desinfestation

Three-month-old tempisque plantlets grown in a greenhouse were provided by the Faustino Miranda Botanical Garden of Tuxtla Gutiérrez Chiapas, a with geographical location 16°45’ N latitude and 93°07’ W longitude. Leaf, stem and root explants were disinfected according to the following protocol: 0.5% (*w*/*v*) captan-agrimycin for 10 min and washed with sterile distilled water three times. Later, the explants were placed in 70% ethanol (*v*/*v*) for 1 min and again washed with sterile water three times. Finally, explants were placed in 9% NaClO (*w*/*v*) with 1 mL of Tween 20 for 20 min and washed with sterile water three times. After the disinfection protocol, explant cuts of approximately 0.5 cm x 0.5 cm were placed in tissue culture flasks with 4.4 g/L Murashige and Skoog medium [63] (MS, Sigma Chemical Co., St. Louis, MO, USA) supplemented with 3% (*w*/*v*) sucrose and 0.25% (*w*/*v*) phytagel^®^ and plant growth regulators as TDZ and 2,4-D.

### 4.2. Callus Induction

For callus induction, a multifactorial design with three factors was used. The first factor was the type of explant with two levels, which were explants of leaves and explants of stems. The second factor was 2,4-D and the third factor was TDZ, both with three levels that were 0, 0.5 and 1 mg/L (Table 6). The explants were maintained in the medium described in the previous section and were incubated in a bioclimatic chamber at 22 ± 1 °C with a photoperiod of 16 h light and monthly subcultures. Each experimental unit contained 4 explants and each treatment was performed in quadruplicate. For the control, the explants were grown without plant growth regulators. The response variables were the percentage of disinfection, the percentage of survival and callus formation index (CFI) obtained with the following formula [12]:

(1) $$\mathrm{CFI}=\frac{n\;G}{N}\;\times \;100$$

where *n* = total callus formed; *G* = average callus score; *N* = total number of cultured explants.

### 4.3. Experiment with UV-B Radiation

For treatments with UV-B radiation, calluses obtained from leaf and stem explants were used. These calluses were subcultured monthly and maintained in MS medium [46] (Sigma Chemical Co., St. Louis, MO, USA), supplemented with 3% (*w*/*v*) sucrose, 0.25% (*w*/*v*) phytagel^®^, 1 mg/L of 2,4-D and 1 mg/L of TDZ. After six months of subcultures from disinfection and in vitro establishment, the calluses were exposed to UV-B radiation using a Holland Phillips TL 20 W/01RS lamp with a main wavelength of 312 nm and irradiance of 4.38 W· m^−2^, directly above the cultures with a distance between the lamp and the callus cultures of 30 cm.

#### Experimental Design for the Effect of UV-B Radiation on Callus Cultures

To evaluate the effect of UV-B radiation on callus culture, a multifactorial experimental design was used. One of the factors was the origin of callus with two levels that were the calluses obtained from leaf explants and the calluses obtained from stem explants, while a second factor was the exposure time with four levels, which were 1, 2, 3 and 4 h of exposure to UV-B radiation (Table 7). For the control, the cultures were grown only with white light and the same exposure periods. Each experimental unit contained 4 calluses of 200 mg each and all treatments were carried out with five repetitions. The entire experiment was carried out for two and four weeks as exposure periods [33]. After the exposure periods to UV-B radiation, the calluses were harvested and processed for analysis of morphology, growth parameters and determination of secondary metabolites.

### 4.4. Fresh Weight and Growth Index

The fresh weight of the callus was recorded immediately after the exposure period (two and four weeks) to UV-B radiation. The fresh callus was completely extracted and placed in sterile Petri dishes; the remains of the medium were removed with a scalpel. Fresh weight was determined using a sensitive analytical balance. The growth of the callus was determined in terms of growth index according to the following equation [64]:

(2) $$\mathrm{Growth}\;\mathrm{index}\;\left(\%\right)=\frac{\mathrm{final}\;\mathrm{fresh}\;\mathrm{weight}\;\left(g\right)-\mathrm{initial}\;\mathrm{fresh}\;\mathrm{weight}\left(g\right)}{\mathrm{initial}\;\mathrm{fresh}\;\mathrm{weight}\;\left(g\right)}\times 100$$

### 4.5. Preparation of Methanolic Extracts

The methanolic extracts of callus were obtained according to the method of [65] with modifications of [66]. After measuring the fresh weight, the samples were frozen and lyophilized in the dark for 24 h. The lyophilized samples were pulverized, and 250 mg was used for extraction in 50 mL of methanol for 3 min under sonication at room temperature, then incubated in a water bath at 50 °C for 30 min. Subsequently, the extraction was continued on a rotary shaker at 100 rpm for approximately 15 h. At the end of this period, the samples were centrifuged at 4000 rpm for 10 min. The supernatant was filtered, and the residue was washed twice with 5 mL of methanol and subsequently filtered again. The combined supernatants were evaporated under reduced pressure and stored as dry extract at −20 °C until use to resuspend in 3 mL of methanol.

### 4.6. Determination of Total Phenol Content

The total phenolic content was determined with Folin–Ciocalteu reagent as described by [67]. Briefly, 100 μL of the methanolic extract of each sample was diluted in 4.2 mL of water with 0.5 mL of Folin–Ciocalteu reagent; after 1 min of mixing at room temperature, the reaction was neutralized with 1 mL of sodium carbonate at 20% (*w*/*v*). The mixture was incubated for 2 h in the dark at room temperature. The absorbance of the resulting blue color was measured at 765 nm using a UV-VIS spectrophotometer (Beckman Coulter DU 730). The total phenolic content was expressed in mg gallic acid equivalents per gram of dry extract of callus using standard curve of gallic acid. The standard curve was obtained by preparing solutions of 0 to 1000 mg/L of gallic acid dissolved in methanol.

### 4.7. Determination of Total Flavonoids Content

The total flavonoid content was estimated using the colorimetric method described by [68]. Briefly, 0.5 mL of the methanolic extract was mixed with 2.8 mL of water, 1.5 mL of 95% (*v*/*v*) ethanol, 0.1 mL 10% aluminum chloride (AlCl_3_) and 0.1 mL of 1 M potassium acetate and incubated in the dark at room temperature for 30 min. The absorbance of the reaction mixture was measured by spectrophotometry (Beckman Coulter DU 730) at 415 nm. The quantification was carried out with respect to the standard curve of quercetin at concentrations of 10 to 90 mg/L. The results were expressed as equivalent milligrams of quercetin per gram of dry extract of callus.

### 4.8. Quantification of Gallic Acid, Quercetin and Kaempferol in Methanolic Extracts by HPLC

The quantification of flavonoids was carried out following the methodology described by [69]. The methanolic extracts were filtered with a millipore membrane filter with a pore size of 0.22 μm and subsequently stored until the tests were carried out. The chromatographic tests were carried out with high-performance liquid chromatography with a UV–Vis detector (Flexar, Perkin Elmer, Waltham, MA, USA) and a Kromasil 100-5-C18 column (4.6 mm × 150 mm, 5 μm, 100 A; SUPELCO, Bellefonte, CA, USA). For the separation of flavonoids, the mobile phase used was acetic acid at 2.0% (solvent A) and methanol (solvent B). For the separation of phenols, the mobile phase used was 2.0% acetic acid (solvent A) and 0.5% acetic acid: acetonitrile 1:1 (*v*/*v*) (solvent B). The injection volume was 20 µL for all samples. Gallic acid, quercetin and kaempferol quantification was performed by correlation with the standard curves of each metabolite. The data were subjected to regression analysis to determine the concentration of each flavonoid or phenol in callus culture.

### 4.9. Statistic Analysis

All the experiments were conducted under a multifactorial experimental design with four or five repetitions for each experimental unit. Simple ANOVA analysis was carried out to clarify the effects of the interactions between the levels of the factors and multifactorial ANOVA to understand the effect of each factor and each level. Their means were compared using the LSD test (*p* ≤ 0.05) with the help of the statistical software Statgraphics Centurion XVII.

## 5. Conclusions

This research is the first report of the disinfection explants for their establishment on in vitro culture, callus induction and quantification of secondary metabolites in tempisque (*Sideroxylon capiri* Pittier). The greatest response in callus induction was with the combination of TDZ and 2,4-D. The UV-B radiation increased callus growth rates. The stress induced for UV-B radiation was an important factor to improve and increase the concentration of quercetin, kaempferol and gallic acid in callus obtained from leaf and stem explants after 4 weeks of exposure. UV-B radiation could be an effective method for the production of secondary metabolites on *Sideroxylon capiri* callus culture. A perspective of this research could be to establish the in vitro propagation protocol of tempisque for its preservation, to contribute to the knowledge and management of native species and to generate adequate strategies for reforestation and sustainable exploitation because this biotechnological aspect has not been reported in this species.

## Figures and Tables

**Table 1:** Effect of time of exposure to UV-B radiation and origin explant on the fresh weight of the callus culture.

Fresh Weight of Callus (mg)							
Exposure Period (Weeks)	Origin of Callus	Exposure Time to UV-B (h/day)					Mean Explant of Origin (LSD = 16.45)
		0	1	2	3	4	
2	Leaf	612.8 ± 9.7 d	618.5 ± 12 d	764.9 ± 21 c	837 ± 23 b	907.3 ± 7 a	748.1 A
	Stem	547.4 ± 15 e	558.2 ± 42 e	563.5 ± 31 e	803 ± 20 b	894 ± 6 a	673.2 B
Average exposure time (LSD = 26.0)		580.1 D	588.3 D	664.2 C	820 B	900.7 A	Interactions (LSD = 36.78)
		0	1	2	3	4	Mean explant of origin (LSD = 49.44)
4	Leaf	781 ± 129 cd	858.2 ± 46 bc	880 ± 64 bc	919.7 ± 43 b	1289.4 ± 107 a	945.8 A
	Stem	847.1 ± 19 bc	940.5 ± 60 b	864.6 ± 25.1 bc	946 ± 17 b	695.8 ± 24 cd	858.8 B
Average exposure time (LSD = 78.18)		814.3 C	899.4 B	872.3 BC	932.9 AB	992.6 A	Interactions (LSD = 110.56)

LSD = least significant difference (*p* ≤ 0.05). Lower case letters indicate significant difference for interactions between factors (simple ANOVA). Capital letters indicate the statistical significance for each level for each general factor (multifactorial ANOVA). Statistical analyses were conducted separately for each exposure period.

**Table 2:** Effect of time of exposure to UV-B radiation and origin explant on the dry weight of the callus culture.

Dry Weight of Callus (mg)							
Exposure Period (Weeks)	Origin of Callus	Exposure Time to UV-B (h/day)					Mean Explant of Origin (LSD = 1.60)
		0	1	2	3	4	
2	Leaf	53.56 ± 3.1 e	55.46 ± 0.8 de	69.63 ± 0.8 c	78.4 ± 0.7 b	83.86 ± 0.8 a	68.18 A
	Stem	32.46 ± 1.2 g	35.13 ± 3.8 fg	36.9 ± 2.7 f	58.93 ± 1.8 d	70.2 ± 2.3 c	46.72 B
Average exposure time (LSD = 2.53)		43.01 D	45.3 D	53.26 C	68.66 B	77.03 A	Interactions (LSD = 3.58)
		0	1	2	3	4	Mean explant of origin (LSD = 3.15)
4	Leaf	63.2 ± 2.8 f	75.5 ± 3.9 de	79.2 ± 5.3 cd	83.1 ± 1.8 bc	149.3 ± 9 a	90.04 A
	Stem	70.7 ± 0.8 e	86.9 ± 4.2 b	77.5 ± 1.9 cde	82 ± 1.2 bcd	81.2 ± 1.3 bcd	79.74 B
Average exposure time (LSD = 4.99)		66.96 C	81.17 B	78.31 B	82.8 B	115.21 A	Interactions (LSD = 7.06)

LSD = least significant difference (*p* ≤ 0.05). Lower case letters indicate significant difference for interactions between factors (simple ANOVA). Capital letters indicate the statistical significance for each level for each general factor (multifactorial ANOVA). Statistical analyses were carried out separately for each exposure period.

**Table 3:** Effect of time of exposure to UV-B radiation and origin explant on the growth index of the callus culture.

Growth Index of Callus (%)							
Exposure Period (Weeks)	Origin of Callus	Exposure Time to UV-B (h/day)					Mean Explant of Origin (LSD = 6.58)
		0	1	2	3	4	
2	Leaf	145.1 ± 4 d	147.4 ±5 d	206 ± 8 c	234.8 ± 9 b	263 ± 3 a	199.245 A
	Stem	118.9 ± 6 e	123 ± 17 e	125 ± 12 e	221± 8 b	257.6 ± 3 a	169.287 B
Average exposure time (LSD = 10.41)		132.04 D	135.33 D	165.67 C	228 B	260.27 A	Interactions (LSD = 14.71)
		0	1	2	3	4	Mean explant of origin (LSD= 19.78)
4	Leaf	212 ± 51 cd	243 ± 19 bc	252 ± 26 bc	268 ± 17 b	415.7 ± 43 a	278.305 A
	Stem	238.8 ± 8 bc	276.2 ± 24 b	245.8 ± 10 bc	278.4 ± 7 b	178.3 ± 10 d	243.526 B
Average exposure time (LSD = 31.27)		225.73 C	259.74 B	248.9 BC	273.16 AB	297.02 A	Interactions (LSD = 44.23)

LSD = least significant difference (*p* ≤ 0.05). Lower case letters indicate significant difference for interactions between factors (simple ANOVA). Capital letters indicate the statistical significance for each level for each general factor (multifactorial ANOVA). Statistical analyses were conducted separately for each exposure period.

**Table 4:** Effect of exposure time to UV-B radiation and source explant on total phenol concentration in callus culture.

Total Phenols (mg Gallic Acid Equivalents/g Dry Weight of Callus)							
Exposure Period (weeks)	Origin of Callus	Exposure Time to UV-B (h/day)					Mean Explant of Origin (LSD = 0.31)
		0	1	2	3	4	
2	Leaf	6.97 ± 0.2 e	7.3 ± 0.3 e	9.43 ± 0.5 bc	8.9 ± 0.6 c	11.2 ± 0.7 a	8.77 A
	Stem	7.07 ± 0.1 e	7.4 ± 0.3 de	7.6 ± 0.34 de	8.2 ± 0.5 d	9.8 ± 0.4 b	8.01 B
Average exposure time (LSD = 0.49)		6.99 C	7.36 C	8.53 B	8.58 B	10.50 A	Interactions (LSD = 0.69)
		0	1	2	3	4	Mean explant of origin (LSD = 0.23)
4	Leaf	8.4 ± 0.1 e	10.4 ± 0.3 cd	10.7 ± 0.3 bc	11 ± 0.1 b	12.2 ± 0.14 a	10.55 A
	Steam	8.5 ± 0.54 e	8.4 ± 0.2 e	9.9 ± 0.2 d	10.5 ± 0.5 bc	11 ± 0.05 b	9.70 B
Average exposure time (LSD = 0.36)		8.46 D	9.42 C	10.35 C	10.78 B	11.62 A	Interactions (LSD = 0.52)

LSD = least significant difference (*p* ≤ 0.05). Lower case letters indicate significant difference for interactions between factors (simple ANOVA). Capital letters indicate the statistical significance for each level for each general factor (multifactorial ANOVA). Statistical analyses were conducted separately for each exposure period.

**Table 5:** Effect of exposure time to UV-B radiation and origin explant on total flavonoid biosynthesis in callus culture.

Total Flavonoids (mg Quercetin Equivalents/g Dry Weight)							
Exposure Period (Weeks)	Origin of Callus	Exposure Time to UV-B (h/day)					Mean Explant of Origin (LSD = 0.23)
		0	1	2	3	4	
2	Leaf	3.11 ±0.07 ef	3.39 ±0.3 ef	3.76 ± 0.17 ef	6.6 ± 0.12 b	7.39 ±0.38 a	4.85 B
	Stem	4.52 ± 0.43 d	5.38 ± 0.3 c	5.79 ± 0.17 c	5.58 ± 0.43 c	6.88 ±0.3 ab	5.63 A
Average exposure time (LSD = 0.37)		3.81 E	4.39 D	4.78 C	6.09 B	7.13 A	Interactions (LSD = 0.52)
		0	1	2	3	4	Mean explant of origin (LSD = 0.12)
4	Leaf	4.71 ± 0.17 h	5.75 ±0.18 g	6.28 ± 0.13 e	7.5 ± 0.05 b	8.32 ±0.09 a	6.5227 A
	Stem	4.22 ± 0.2 i	6.1 ±0.14 fg	6.13 ± 0.18 ef	6.92 ± 0.18 d	7.24 ±0.14 c	6.10749 B
Average exposure time (LSD = 0.19)		4.46 E	5.88 D	6.20 C	7.23 B	7.78 A	Interactions (LSD = 0.27)

LSD = least significant difference (*p* ≤ 0.05). Lower case letters indicate significant difference for interactions between factors (simple ANOVA). Capital letters indicate the statistical significance for each level for each general factor (multifactorial ANOVA). Statistical analyses were carried out separately for each exposure period.

**Table 6:** Multifactorial experimental design to evaluate the response of the type of *Sideroxylon capiri* Pittier explant and the type of growth regulator on the callus formation index (CFI).

Treatment	Explant Type	Plant Growth Regulator (mg/mL)	
		2,4-D	TDZ
1 *	Leaf	0.0	0.0
2	Leaf	0.0	0.5
3	Leaf	0.0	1.0
4	Leaf	0.5	0.0
5	Leaf	0.5	0.5
6	Leaf	0.5	1.0
7	Leaf	1.0	0.0
8	Leaf	1.0	0.5
9	Leaf	1.0	1.0
10 *	Stem	0.0	0.0
11	Stem	0.0	0.5
12	Stem	0.0	1.0
13	Stem	0.5	0.0
14	Stem	0.5	0.5
15	Stem	0.5	1.0
16	Stem	1.0	0.0
17	Stem	1.0	0.5
18	Stem	1.0	1.0

* Control treatment, explants grown without plant growth regulator.

**Table 7:** Experimental design to evaluate the explant of origin of the callus and the exposure time to UV-B radiation on morphology, growth parameters and secondary metabolites.

Treatment	Origin of Callus	Exposure Time to UV-B (h/day)
1 *	Leaf	0
2	Leaf	1
3	Leaf	2
4	Leaf	3
5	leaf	4
6 *	Stem	0
7	Stem	1
8	Stem	2
9	Stem	3
10	Stem	4

* Control treatment, grown without UV-B radiation.

## Data Availability

Not applicable.

## Abbreviations

TDZ	Thidiazuron
2,4-D	2,4-dichlorophenoxyacetic acid
CFI	Callus formation index
ROS	Reactive Oxygen Species
UVR8	Ultraviolet resistance locus 8
COP 1	Constitutive photomorphogenic 1
HY5	Elongated Hypocotyl 5
DELLA	(aspartic acid–glutamic acid–leucine–leucine–alanine) proteins
PIF	Phytochrome Interacting Factors
Aux/AIA	Auxin/indole-3-acetic acid
